# Detection of cancer before distant metastasis

**DOI:** 10.1186/1471-2407-13-283

**Published:** 2013-06-13

**Authors:** Frank AW Coumans, Sabine Siesling, Leon WMM Terstappen

**Affiliations:** 1 Medical Cell BioPhysics group, MIRA Institute, University of Twente, Room CR4437, Hallenweg 23, 7522 NH, Enschede, The Netherlands; 2 Department of Research, Comprehensive Cancer Center the Netherlands, PO Box 19079, 3500 DB, Utrecht, The Netherlands; 3 Department of HTSR, MIRA institute for Biomedical Technology and Technical Medicine, University of Twente, Enschede, the Netherlands

**Keywords:** Metastasis, Diagnostic imaging, Modeling, Tumor size, Circulating tumor cells

## Abstract

**Background:**

To establish a distant metastasis (DM) cells must disseminate from the primary tumor and overcome a series of obstacles, the metastatic cascade. In this study we develop a mathematical model for this cascade to estimate the tumor size and the circulating tumor cell (CTC) load before the first metastasis has formed from a primary breast cancer tumor.

**Methods:**

The metastatic cascade is described in discrete steps: 1. local tumor growth; 2. dissemination into circulation; 3. survival in circulation; 4. extravasation into tissue; and 5. growth into a metastasis. The model was built using data and relationships described in the literature to predict the relationship between tumor size and probability of distant metastasis for 38715 patients with surgically removed T_X_N_X_M_0_ primary breast cancer from the Netherlands Cancer Registry. The model was calibrated using primary tumor size, probability of distant metastasis and time to distant metastasis for 1489 patients with stage T_1B_N_X_M_0_ (25% of total patients with T_1B_N_X_M_0_). Validation of the model was done with data for all patients.

**Results:**

From the time to distant metastasis of these 38715 breast cancer patients, we determined a tumor doubling time of 1.7 ± 0.9 months. Fitting the data for 25% of T_1B_ patients estimates a metastatic efficiency of 1 metastasis formed per 60 million disseminated tumor cells. Validation of the model to data of patients in all T-stages shows good agreement between model and epidemiological data. To reduce the 5-year risk of distant metastasis for T_X_N_X_M_0_ from 9.2% to 1.0%, the primary tumor needs to be detected and removed before it reaches a diameter of 2.7 ± 1.6 mm. At this size, the model predicts that there will be 9 ± 6 CTC/L blood.

**Conclusions:**

To reduce the rate of distant metastasis in surgically treated T_X_N_X_M_0_ breast cancer to 1%, imaging technology will need to be able to detect lesions of 2.7 mm in diameter or smaller. Before CTC detection can be applied in the early disease setting, sensitivity will need to be improved by at least 15-fold and combined with technology that minimizes false positives.

## Background

The majority of deaths from cancer are due to distant disseminated disease rather than the primary tumor [1]. While metastases are often discovered years after surgical removal of the primary tumor, probably at least one metastasis was already present at the time of surgery. Understanding of the formation of distant metastasis (DM) is crucial for the reduction of the recurrence rate. For the successful colonization of a secondary site a cancer cell must complete a series of steps to become a clinically detectable lesion, baptized the metastatic cascade [2-5]. This cascade is an inefficient process, generating metastasis by sending large numbers of malignant cells into the circulation [6,7]. The number of cells disseminated and the efficiency of metastasis formation contribute to the probability that a metastasis has formed. Assays for enumeration of circulating tumor cells (CTC) in blood can provide the number of disseminated cells. The CTC are the new seeds of a tumor, and as such provide an opportunity to estimate the metastatic efficiency. Due to metastatic inefficiency, the presence of CTC does not imply that metastases already exist. Identification and enumeration of CTC at or before the diagnosis of the primary tumor may allow estimation of the probability of DM being present and thus help identify patients who will benefit from more intensive adjuvant therapy after surgical removal of the primary lesion. Here we developed a model for the metastatic cascade and estimated the sensitivity needed for imaging and CTC enumeration to detect a primary tumor before it has formed DM.

## Methods

### Distant metastasis statistics

The probability of DM and time to DM was determined based on patients selected from the population based Netherlands Cancer Registry (NCR, http://www.iknl.nl). Specially trained registrars access the patient files to gather data regarding patient, tumor characteristics, and treatment for all malignancies in all hospitals in the Netherlands. Tumor size is determined by specialized pathologists. From the registry, we selected women who were diagnosed between 2003 and 2006 with pathological stage T_1A_N_X_M_0_-T_2_N_X_M_0_ primary invasive breast cancer, being 25 years or older at time of diagnosis and had mastectomy or breast conserving surgery. Patients were excluded if any evidence of residual tumor was found after surgery. Patients were followed until at least five years after diagnosis and both occurrence and date of DM were registered. Time between diagnosis and occurrence of DM is indicated with the mean and standard deviation. Probability of a DM within the five years of follow-up and the 95% confidence interval of DM were determined by Poisson statistics. The study protocol was approved by the NCR Privacy Council and assured that all necessary consent from the patients were obtained.

### Parameter fitting

A numerical model for the development and detection of DM was developed and tested in Matlab 2009a (Mathworks, Natick, MA). The two essential elements in the model are the number of cells disseminated into circulation with a certain time interval and the probability of metastatic success of each cell. The number of metastases formed is described by a binomial distribution, which is approximated by applying the Poisson limit theorem. The resulting cumulative distribution function was compared to a pseudo random number generated by Matlab. The time to DM in the model was defined as the time between removal of the primary tumor and the time when the first DM reaches 8 mm in diameter (T_1B_). The mean and standard deviation of the doubling time was determined from the time to DM. CTC concentration was fit to available literature values; patients with metastatic disease have 3.0 CTC/mL (5–95 percentile: 0.02-417 CTC/mL) [8-11], and patients with early stage breast cancer have CTC at a mean concentration of 0.03 CTC/mL (range of estimates 0.01-0.05 CTC/mL) [12-15].

The product of dissemination rate and metastatic efficiency was fit to the probability of DM for 25% of patients with stage T_1B_, running 10,000 iterations and randomizing doubling time for each iteration. Stage T_1B_ was selected for fitting because it is the smallest frequently discovered tumor, typically discovered when it reaches a diameter of 8 mm. After fitting the data on a subset of T_1B_ patients we validated the model by comparing predicted and actual probability of DM for all patients grouped by T-stage (T_1A_-T_2_).

## Results

### Model for formation of distant metastasis

**Figure 1:**
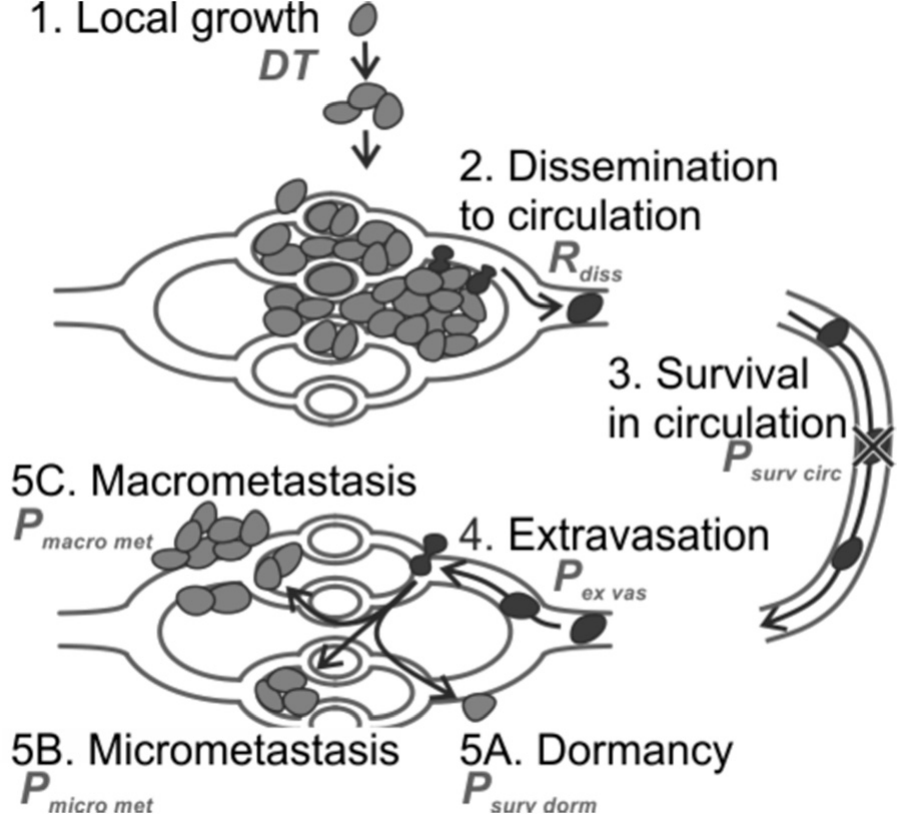
**Steps in the metastatic cascade.** Parameters related to each step are shown near the description of each step. Cells in tissue are indicated in light gray, cells in blood or in transition between blood vessel and tissue are indicated in black. Local growth (step/equation 1) is needed to provide sufficient disseminated cells. Steps 2–5 are typically referred to as the metastatic cascade (equations S3-S5, S8). Step 5 has multiple outcomes, with cells surviving, but not replicating (5A), briefly or slowly replicating (5B) or rapidly replicating (5C).

The steps in the metastatic cascade are summarized in Figure 1. 1). A tumor grows locally. 2). Cells disseminate from the primary tumor. 3). The tumor cells that ultimately survive in the circulation. 4). Arrest of tumor cells in the microcirculation of an organ and potential extravasation into the surrounding tissue. The extravasated tumor cells can either 5A). Survive as a singular dormant cell, 5B). Form a micro metastasis, or 5C). Grow into a macro metastasis. While it is unknown what triggers a primary tumor to start shedding cells into the blood stream, this shedding starts well before the primary tumor is detectable by current imaging techniques [16-18]. We developed a mathematical model for the formation of metastasis using relationships described in literature for each of these steps. Steps 3,4 and 5C together are the probability that a disseminated cell forms a macro metastasis, i.e. the metastatic efficiency (*γ*_*metastatic*_).

In the model the following conventions are applied: the number of cells is described with N, the dissemination rate with R, the diameter with D, the doubling time with DT and other constants with C. Subscripts are used to differentiate different N. The formation of distant metastasis is described with functions for the steps, depicted in Figure 1, see Additional file 1: Supplemental S1 for a more elaborate derivation:

1. *Local growth*: In a comparison of functions typically used to describe tumor growth (exponential, Gompertz or logistic), the logistic function fit best [19], equation 1:

(1) $$\begin{array}{ll} N_{\mathrm{mass}}\left(t\right) & =\frac{N_{\max}}{{\left[1+\left(N_{\max}^{1/4}-1\right)e^{-\ln\left(2\right)\;t/4\;\mathrm{DT}}\right]}^{4}}\\ & \approx \frac{N_{\max}}{{\left[1+{\left(N_{\max}e^{-\ln\left(2\right)\;t/\mathrm{DT}}\right)}^{1/4}\right]}^{4}} \end{array}$$

Gompertz and logistic functions have a slowing growth rate as the tumor reaches a maximum size *N*_*max*_ (N = number of tumor cells) at a certain time (*t*). *N*_*max*_ is typically chosen at 10^12^ cells/1 kilogram [19]. We assume metastases grow according to equation 1. Changes in growth rate due to occurrence of growth enhancing mutations or due to chemo or hormonal therapy are not explicitly considered in any of the growth models.

2. *Dissemination to circulation*: The relationship between tumor diameter (*D*_*mass*_) and the number of disseminated cells (*N*_*diss*_) is assumed linear and is derived from murine data comparing CTC counts to the diameter of the primary tumor [20-23]. To derive the diameter of the lesion from the number of cells (*N*_*mass*_) we assumed a spherical lesion, cells into which disseminates the bloodstream at a rate (*R*_*diss*_):

(2) $$R_{\mathrm{diss}}=C_{\mathrm{diss}}\cdot D_{\mathrm{mass}}$$

*3,4,5C. Formation of a metastasis:* The relationship between the number of cells injected into the circulation and the number of macroscopic metastases is linear [24-26], with a slope *γ*_*metastatic*_ the metastatic efficiency. We now find the total number of metastases:

(3) $$N_{\mathrm{total}\;\mathrm{macro}\;\mathrm{met}}=\int \gamma_{\mathrm{metastatic}}\cdot R_{\mathrm{diss}}\mathrm{dt}$$

In this equation, the number of metastases formed is equal to the metastatic efficiency times the total number of cells disseminated from the tumor. The rate of cell dissemination (*R*_*diss*_) is measurable by detecting the number of CTC, while the metastatic efficiency (γ_metastatic_) may be measurable either by genotyping these CTC or the primary tissue.

Below we listed the assumptions of the model with their impact:

1. Growth of the primary tumor is described by a logistic function. Studies that determine the growth functions do so on relatively large tumors, Table 1. The shape of the growth function when the tumor consists of only a few cells in size is not known.

2. A single malignant breast cell with a size of 10^-6^ mm^3^, starts to grow and disseminate tumor cells into the blood. Although it is unlikely that a tumor starts dissemination from a size of one cell, the starting size only marginally affects the metastatic efficiency. For example the estimate for metastatic efficiency is increased by 1%, if dissemination does not start immediately, but starts once the tumor reaches a size of 0.1 mm (1000 cells).

3. Each of the disseminated cells has the same probability of forming a distant metastasis (DM). It is likely that only a limited number of cells are capable of metastasizing, for example because part of disseminated cells are not viable, or do not have the features needed for metastasis. The estimated metastatic efficiency is the average efficiency. If only 1% of cells are capable of metastasizing, the metastatic efficiency of those cells would be 100 times larger than our estimate over the total number of cells. This assumption does not affect the fit parameters, but could result in enhanced metastatic efficiency of cells disseminated from metastases.

4. The probability of forming a DM is constant over time, i.e. tumor evolution is not considered. While we expect that metastatic efficiency increases over time, the literature does not describe quantitative data for such evolution. If tumor evolution is rapid, our estimated metastatic efficiency is effectively fit to the time-period just before tumor discovery.

5. The probability of forming a DM and the dissemination rate are not dependent on the growth rate of the primary tumor. Considering the high incidence of recurrence within the first 5 years compared to years 6–15, metastatic efficiency or dissemination rate probably are smaller for slower growing tumors. If, for example, 75% of recurrences are found in the first five years (doubling time < 3 months), 20% of recurrences in years 5–15 (doubling time 3–9 months), and 5% of recurrences in years 15–25 (doubling time 9–15 months), the metastatic efficiency/dissemination rate for a 0–3 month doubling time would be approximately 6-fold higher than a 4–9 month doubling time and approximately 24-fold higher than a 10–15 month doubling time.

6. Both the rate of dissemination and the probability of distant metastasis formation are independent of the cancer type. We need to make this assumption because (1) data on CTC concentrations versus cancer type is not available, and (2) data on cancer types for the patients in our data set is not available. In another study, the five year risk of recurrence for patients with triple negative breast cancer was estimated to be 2.6 fold higher than for patients with other breast cancers [37]. This implies that the product of dissemination rate and metastatic efficiency needs to be approximately 2.6 fold higher for patients with triple negative breast cancer.

7. Disseminated cells are not temporarily ‘stored’ in the bone marrow (tumor dormancy). In the model we assume the transit from primary tumor to metastatic site occurs within days. Temporary storage (dormancy) of cells in the bone marrow at the metastatic site, would result in a delay in the start of growth, and thus in an underestimation of the doubling time. For long delays the recurrence would likely be pushed outside the 5-year window, and for small delays the doubling time is marginally affected. For example, if a typical delay would be four months, the previous doubling time estimate of 1.7 months would become 1.5 months.

8. All primary tumors are removed once they reach a predefined (constant) diameter corresponding to the median size at each stage. This assumption has a negligible impact on growth rate due to the small range of sizes within each T-stage.

9. In case of DM at least one metastasis was formed prior to surgery. This assumption means that all metastases originate from the primary tumor, and implies no patients formed a second primary tumor. This assumption leads to an overestimation of the rate of recurrence, and thus an overestimation of the metastatic efficiency. For example, if 10% of DM were misclassified and actually a second primary, the risk of recurrence is reduced by 10% and the metastatic efficiency is reduced by 10%.

10. The probability of DM is defined as the probability that at least one metastasis was present at the time of surgery, continues to grow and is discovered once it has reached a size of 8 mm. Changing the size at which a tumor is discovered affects the estimated growth rate. For example, if the typical tumor is discovered at a size of 15 mm, the estimated doubling time is 1.5 months instead of 1.7 months.

11. For fitting of the model, the primary tumor is detected when it reaches a size of 8 mm (stage T_1B_). This assumption has negligible impact on growth rate due to the small range of sizes within each T-stage.

12. For validating the model, the primary is detected when it reaches the median size representative of each T_X_ stage*.* This assumption has negligible impact on growth rate due to the small range of sizes within each T-stage.

13. Cardiac output is 5 L/minute. The cardiac output is used to convert the CTC concentration to the dissemination rate. A different cardiac output only affects the dissemination rate and metastatic efficiency. A cardiac output of for example 6 L/minute would reduce the estimated dissemination rate by 17%, but increase the estimated metastatic efficiency by 20%.

14. Dissemination rate is proportional to tumor diameter. Literature values in murine models [20,23,38-42] suggest a linear relationship between dissemination rate and tumor diameter. However, most of these determine three data points. The model does not fit the epidemiological data if we assume the dissemination rate to be proportional to the tumor surface area, or the number of cells in the tumor.

**Table 1:** Human breast cancer values for doubling time

		**Tumor**	**Doubling time (months)**	
**Publication**	**N**	**size (mm)**	**Median**	**Range**
Lundgren 1977 [27]	13	N/A	6.9	1.4 – 13.0 ^a^
Heuser 1979 [28]	32	14	10.6 ^b^	3.6 – 31.0 ^a^
Von Fournier 1980 [29]	100	17	6.6 ^b^	2.1 – 20.6 ^c^
Galante 1981 [30]	196	N/A	2.0	N/A
Tabbane 1989 [31]	75	N/A	3.8	0.5 – 25.3 ^a^
Kuroishi 1990 [32]	142	N/A	5.7 ^b^	0.4 – 42.4 ^a^
Spratt 1993 [19]	448	9	8.5	5.0 – 15.7 ^d^
Peer 1993 [33]	236	>10	5.0	1.5 – 17.1 ^e^
Tilanus-Linthorst 2005 [34]	47	12	2.0 ^b^	0.4 – 18.6 ^a^
Weedon-Felkjaer 2008 [35]	969	15	3.1	1.0 – 9.6 ^d^
Millet 2011 [36]	37	9	11.2 ^b^	−222.9 – 146.6 ^a^

N/A: not available, ^a^ minimum-maximum, ^b^ mean, ^c^ 2.5-97.5 percentile, ^d^ 25-75 percentile, ^e^ 68% confidence interval.

### Distant metastasis statistics

**Figure 2:**
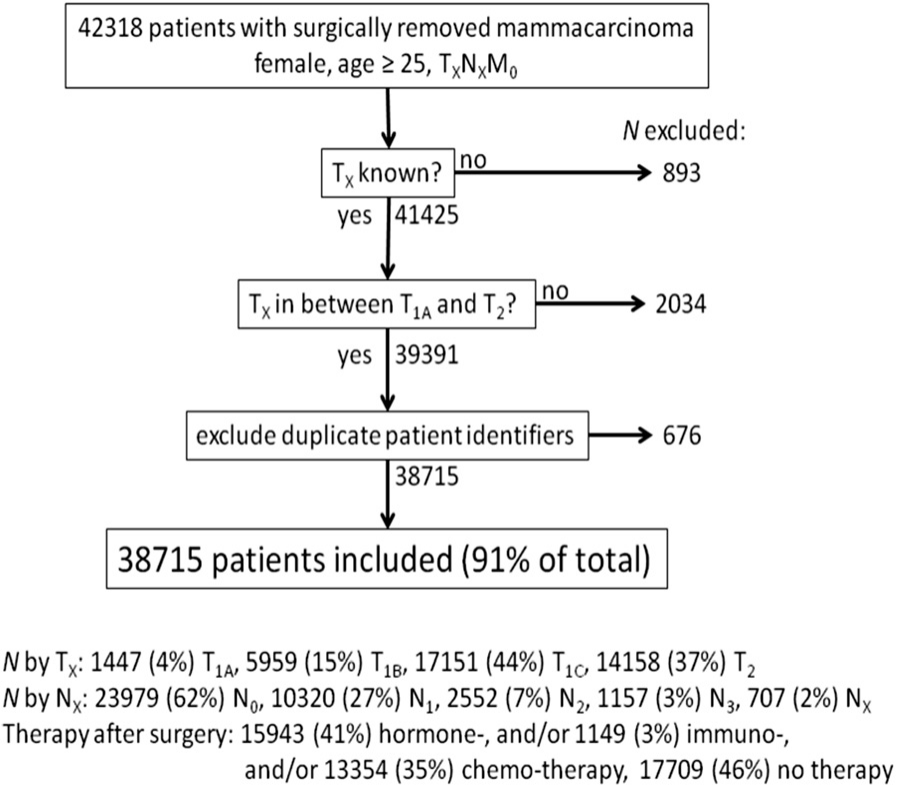
**Exclusion diagram.** Of 42318 patients included by the initial search criteria, 3603 (9%) were excluded for the reasons detailed in the diagram. The distribution of patients by T-stage, node (N) status and therapy after surgery are shown for the 38715 included patients.

**Figure 3:**
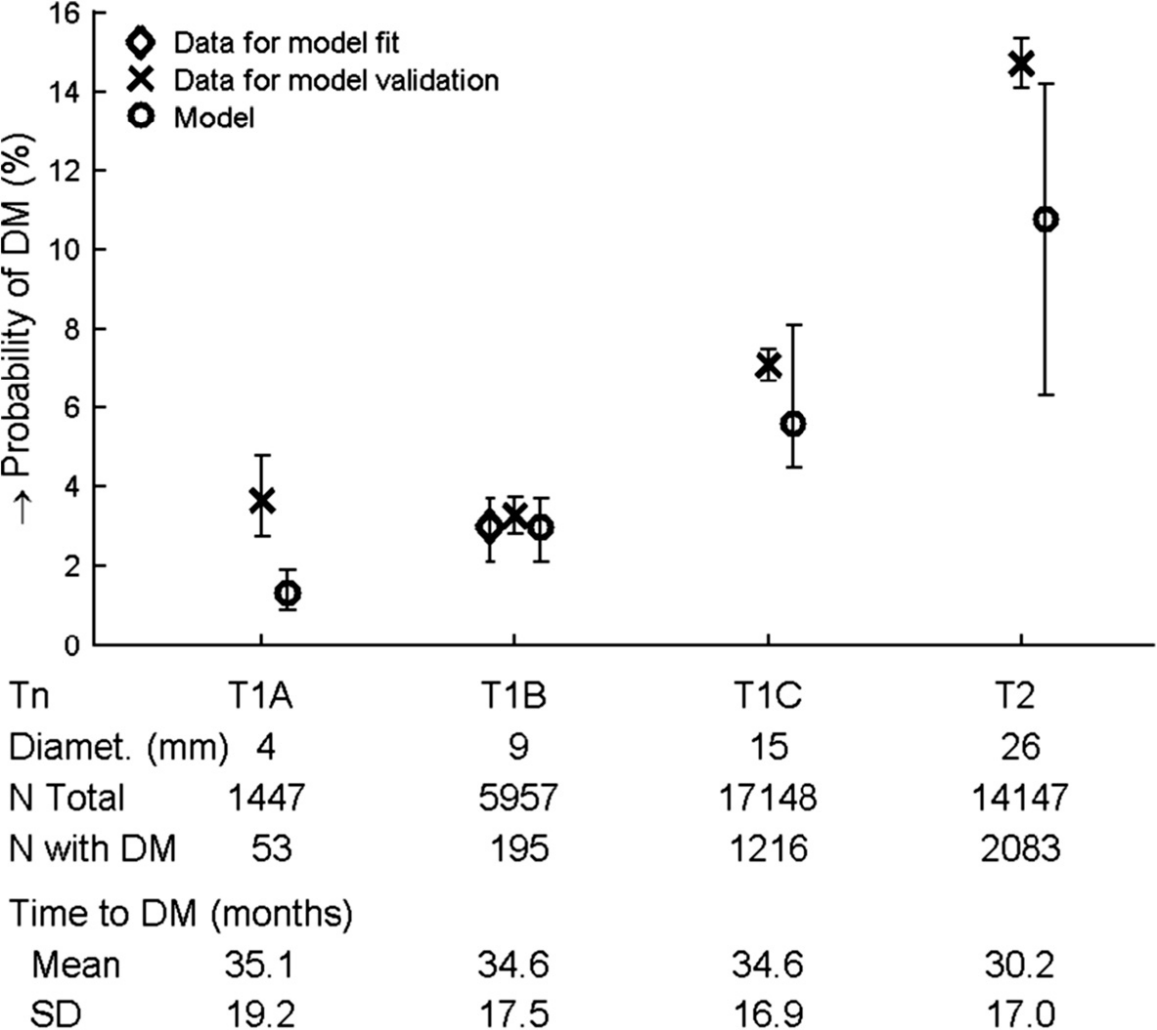
**Probability of distant metastasis by T-stage for patients who are N**_**X**_**M**_**0**_**.** Total *n* = 38715, number of DM n = 3550. Whiskers indicate 95% confidence intervals determined using Poisson statistics. Below the T-stage further statistics are shown, including median diameter in mm, number of patients included, number of patients with a DM as well as mean and standard deviation of time to DM. The model was fit to 25% of the data for T_1B_ tumors. In this subset of the data, there were 42 occurrences of DM in 1489 patients (2.8%) compared to 195 occurrences in 5957 patients (3.3%). Probability of DM for T_1A_, T_1C_ and T_2_ was predicted using the model fit to T_1B_ data.

Of 42318 patients matching our search criteria, 38715 (91%) patients were included, see Figure 2 for exclusion details. The probability of DM after surgery and the time to DM from stage T_1A_ – T_2_ invasive breast carcinoma patients, without known metastases at time of diagnosis (N_X_M_0_) was determined and shown in Figure 3. Three thousand five hundred and fifty patients (9.2%) developed DM within five years after surgery. The overall time to DM was 32.0 ± 17.2 months. Variation in mean time to DM between different T-stages is small (range 30.2 – 35.1 months). 95% of detected primary tumors are 5 mm or larger (median 17 mm). Information of both T-stage and diameter of the primary was available for 33876 (88%) patients in our database. We assumed that the 12% of patients with known T-stage, but without data for the diameter of the primary tumor had the same diameter as the 88% of patients with this data. Probability of DM, time to DM and median size by stage was used to fit the model (equation 3) to the clinical data for 25% of patients with T_1B_ primary tumors. Of 1493 T_1B_ patients used to fit the model, 42 developed DM (2.8%), while in the full data set, 195 of 5975 (3.3%) T_1B_ patients developed DM. We compare the data from the model fit with values reported in the literature for both human and murine studies in Table 2, with more detailed information in Additional file 1: Table S1.

**Table 2:** Metastatic cascade parameter estimates from our model, human and murine studies

**Parameter**	**Model**	**Human**	**Murine**
Doubling time (months)	1.7 ± 0.9	5.7 (2.0-11.2)	
Dissemination rate (CTC/h/g)	280 (90–470)	3.1 · 10^3^ (90–78 · 10^3^)	1.0 · 10^5^ (1.5 · 10^-1^-8.7 · 10^6^)
Metastatic efficiency	1.7 · 10^-8^ (1.3 · 10^-8^-4.2 · 10^-8^)		7 · 10^-5^ (1 · 10^-6^-6 · 10^-3^)

Literature values are the median of all estimates with the range of estimates in parenthesis. Detailed data for each publication is accessed through Additional file 1: Table S1.

### Tumor doubling time

From the time to DM of the 38715 breast cancer patients, we determined a doubling time (*DT*) of 1.7 ± 0.9 months. Human values for *DT* are estimated by fitting a growth model to imaging data reported in the literature, see Additional file 1: Table S2. The median *DT* is estimated at 5.7 months (range 2.0 to 11.2 months) in these reports.

### Formation of metastases and dissemination rate

The probability of forming a metastasis is primarily determined by (1) the number of cells entering the circulation and (2) the probability that each of these cells forms a metastasis (γ_metastatic_). The number of cells entering the circulation is a function of the dissemination rate (*R*_diss_) and the elapsed time, which is affected by the tumor doubling time (*DT*). For a given *DT*, the probability to form a metastasis before surgery is determined by the product of γ_metastatic_ and *R*_diss_. We derived γ_metastatic_ from this product by assuming that R_diss_ is directly related to the CTC concentration, as described in Additional file 1: Supplemental S2. The CTC concentration reported for primary breast cancer before surgery is 0.03 CTC/mL (range 0.01-0.05 CTC/mL [12-15]). We now find a dissemination rate for an 8 mm tumor of 280 CTC/h · g tumor (range 90–470 CTC/h · g tumor) and a metastatic efficiency of 1.7 · 10^-8^ metastases formed per disseminated cell (range 1.3 · 10^-8^-4.2 · 10^-8^), or approximately 60 million disseminated cells per formed macrometastasis.

For comparison, the dissemination rate can be estimated from two human studies which determined the CTC concentration in the efferent vein of colorectal and renal cancer, Table 3, with a median estimate of 3,100 CTC/h · g tumor, and an estimated range of 90–78,000 CTC/h · g. Metastatic efficiency has not yet been estimated in humans. In murine models, the dissemination rate determined by various techniques spans a wide range of nearly 7 orders of magnitude, Table 2 and Additional file 1: Table S1. The median estimate is 1.0 · 10^5^ CTC/h · g (range 0.15 to 8.7 · 10^6^ CTC/h · g). Metastatic efficiency has been determined either from the number of macro metastases formed from injection of a known number of malignant cells, or by observing the individual probabilities in the metastatic cascade by means of intra-vital video microscopy (IVM, [43]). Methods, which determined the metastatic efficiency from injection of a known number of cells, estimated γ_metastatic_ at 0.005% (range 0.0001-6%), Additional file 1: Table S3. From the IVM studies we find a comparable γ_metastatic_ of 0.011%, primarily caused by the low probability of extravasated cells to form a macro metastasis, Additional file 1: Supplemental S3.

**Table 3:** Human values for dissemination rate

**Publication**	**Cancer**	**N**	**Diameter (mm)**	**Disseminated cells **^**a**^	**R**_**diss **_**(CTC/h · g)**^**b**^
Glaves 1988 [44]	Renal	15	5-10	890	78 · 10^3^
Wind 2009 [45]^c^	Colon	13	4.1 ± 1.4	0.05	90
Wind 2009 [45]^d^	Colon	18	5.0 ± 2.3	3.2	3.1 · 10^3^

^a^ mean CTC/mL tumor efferent blood, ^b^ for spherical tumor, ^c^ laparoscopic surgery, ^d^ open surgery.

CTC in a metastatic patient are present at a concentration of 3 CTC/mL of blood [11], however a 100-fold lower concentration is detected in patients before surgery. In our model the number of circulating tumor cells is linked to the total tumor size. While the total tumor size of all lesions is larger for a metastatic patient, the difference is not sufficient to cause such a high change in CTC concentration. To achieve the higher CTC concentration post-surgery, we increased the dissemination rate by 25-fold for all metastatic lesions. We could also achieve this CTC concentration by increasing the metastatic efficiency 10,000-fold. Either scenario, or a combination, is conceivable, since a cell that has completed the metastatic cascade has proven to be capable of dissemination into the circulation and of formation of a metastasis.

### Sensitivity needed for radiographic imaging and CTC detection to detect a tumor before it gives rise to metastasis

**Figure 4:**
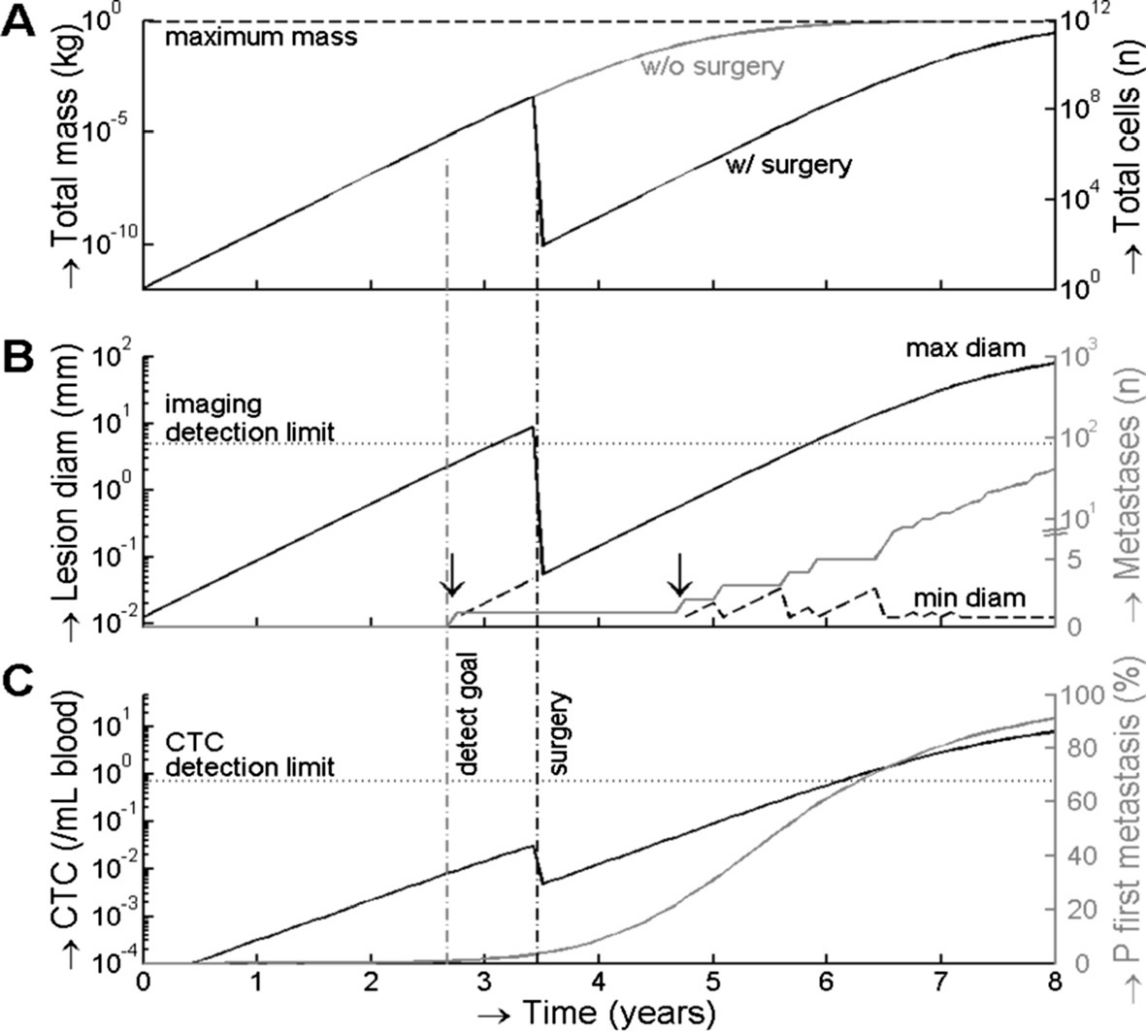
**Application of the model to estimate the technology requirements for radiographic imaging and CTC detection to reduce the probability of distant metastasis to below 1%.** Panel **A** shows the time needed to develop a tumor mass and the number of tumor cells for a stage T_1B_ tumor removed by surgery (black line) or not removed by surgery (gray line). Panel **B** shows the maximum (black solid line) and minimum size (black dashed line) of the primary tumor and/or metastasis and the number of metastasis in gray. Minimum size is only shown when 2 or more lesions exist, the formation of the second lesion before and after surgery are indicated with a vertical black arrow. Panel **C** shows the development of the CTC concentration in black, and the cumulative probability that a first metastasis has occurred in gray. The black vertical dashed line indicates the time at which the primary tumor is surgically removed in our model. The cumulative probability is used to determine the detection goal (vertical dashed gray line); the time when the primary tumor needs to be removed to reduce 5 year recurrence from 9% (from all T_X_N_X_M_0_) to 1%. This line intercepts with a CTC concentration of 9 CTC/L of whole blood and a lesion size of 2.7 mm, and represent the required sensitivities for radiographic imaging and CTC detection.

The model was used to predict the technology needs for detection of tumors before metastasis can occur. The values used for the model are provided in Table 2. In Figure 4 an example is shown of a T_1B_ breast tumor. Panel A shows the development of the total tumor mass and the tumor cell number per equation 1. The black line represents the case for which the tumor is surgically removed and the gray line the case for which the tumor is not removed. In panel B, the solid black line shows the maximum diameter of the tumor. This diameter is important for detection of a tumor by an imaging method. In this case, the T_1B_ tumor is detected when it reaches 8 mm, 3.4 years after its inception, and is surgically removed. If an imaging system is employed to detect all lesions in a patient, it must be capable of identifying the smallest lesions. The dashed line in panel B shows the diameter of the smallest lesion at times multiple lesions exist. In Figure 4, the tumor has seeded a metastasis 2.8 years after initiation of the tumor. At the time of surgery this lesion has a diameter of 70 μm; undetectable by imaging. The total number of metastases is shown in a solid gray line on the secondary y-axis. The number of metastases is relatively stable from 3.4 years to ~5.5 years after surgery, but rapidly increases after 6 years because the metastasis has become sufficiently large to make formation of new metastases sufficiently probable. Panel C shows the CTC concentration in solid black and the probability of forming the first DM is solid gray on the secondary y-axis. To reduce the probability of DM from 9.2% in the patients included in our study to 1%, the tumor needs to be detected by the time it reaches 2.7 ± 1.6 mm, or when the CTC concentration is 9 ± 6 CTC/L whole blood.

## Discussion

The most effective therapy to treat breast cancer is to surgically remove the primary tumor before it has formed a distant metastasis (DM). Unfortunately the technology available to detect the presence of DM at the time of diagnosis cannot accurately make this determination and a large portion of patients receiving adjuvant therapy do not benefit from this therapy whereas others could have benefitted from adjuvant therapy they did not receive. To identify those patients at risk for DM the traditional TNM-classification has been complemented with differentiation grade, peri-tumor vascular invasion, estrogen, progesterone, Her2neu receptor expression and more recently through molecular characterization of the tumor [46-51]. Although improvement in the risk assessment helps to identify the patients that need additional therapy after surgical removal of the primary tumor, detection of the actual presence of tumor cells beyond the primary tumor is preferred. Indeed the presence of micrometastases in bone marrow [52,53] and tumor cells in blood [12-14,54] of breast cancer patients have been associated with an increased risk for disease recurrence, but have not become part of clinical practice partly because the current technology lacks sufficient sensitivity and specificity. The observations that CTC have been detected in patients years after a diagnosis and treatment of breast cancer with curative intent further challenges the technology to identify those CTC characteristics that predict imminent relapse [55,56].

To identify the basic requirements for detection of DM we have modeled the probability that a DM has been formed prior to surgery. Three key components of this probability are the tumor doubling time (*DT*), the rate of tumor cell dissemination (*R*_*diss*_), and the probability of successful completion of the metastatic cascade (*γ*_*metastatic*_). Rate of dissemination can be determined from the CTC concentration values reported in literature [8-15]. Here we combined literature values with clinical data from the NCR to obtain estimates for *DT*, and (*γ*_*metastatic*_) for patients. Using this model, we predicted the sensitivity needed for radiographic imaging and CTC enumeration for the detection of a primary tumor before DM formation has occurred.

The major assumptions in the model are:

1) Metastatic efficiency and dissemination rate are not dependent on doubling time. Considering the high incidence of recurrence within the first 5 years compared to years 6–15, metastatic efficiency or dissemination rate are probably smaller for slower growing tumors. If, for example, 75% of recurrences are found in the first five years (doubling time < 3 months), 20% of recurrences in years 5–15 (doubling time 3–9 months), and 5% of recurrences in years 15–25 (doubling time 9–15 months), the metastatic efficiency for 0–3 month doubling time would be approximately 6-fold higher than for 3–9 months, and approximately 24-fold higher than for 9–15 months. A shorter doubling time reduces the probability of DM; the tumor has a shorter time to form a metastasis before it is large enough to be discovered. However, a reduction of probability of DM by an x-fold shorter doubling time is negated by a x^½^-fold higher metastatic efficiency. A 6 fold increase in metastatic efficiency and/or dissemination rate for a fourfold shorter doubling time would mean that the faster growing tumor has a higher probability of DM.

2) The rate of dissemination and metastatic efficiency are independent of cancer type. We need to make this assumption because 1.) We lack data on CTC concentrations versus cancer type, and 2.) We lack data on cancer types for the patients in our data set. In another study, the five year risk of recurrence for patients with triple negative breast cancer (11% of total) was estimated to be 2.6 fold higher than for patients with other breast cancers (89% of total) [37]. To assess the impact of a subtype with high risk of recurrence, we implemented a subgroup of 11% of patients with 2.6 fold higher product of metastatic efficiency and dissemination rate than the other 89%, while the average metastatic efficiency was held constant. The estimated detection limits did not change due to a higher metastatic efficiency nor to a higher dissemination rate.

3) The metastatic efficiency does not evolve over time. While we expect that metastatic efficiency actually increases over time [16], we lack data describing such evolution. The high relative probability of distant metastasis formation just before tumor discovery implies that the estimated metastatic efficiency also applies to the period just before tumor discovery. To obtain a fit between the CTC data in early stage patients and in metastatic patients, we applied a single increase in the dissemination rate of 25-fold, or an increase in the metastatic efficiency of 10,000-fold. Our rationale was that the metastatic cell has become efficient at disseminating and/or metastasizing due to natural selection by the metastatic cascade and has thus become genetically more prone to formation of new metastases [57,58]. We recognize that it is equally feasible that such evolution occurs more gradually.

4) The transit from primary tumor to metastatic site is instant. Temporary storage of cells in the bone marrow, or temporary dormancy at the metastatic site, would result in a delay in the start of growth, and thus in an underestimation of the doubling time. For long delays the recurrence would likely be pushed outside the 5-year window, and for small delays the doubling time is marginally affected. For example, if we assume a typical delay of four months, the previous doubling time estimate of 1.7 months would become 1.5 months.

5) The probability of DM is defined as the probability that at least one metastasis was present at the time of surgery, continues to grow and is discovered once it has reached a size of 8 mm. Changing the size at which a tumor is discovered affects the estimated growth rate. For example, if the typical tumor is discovered at a size of 15 mm, the estimated doubling time is 1.5 months instead of 1.7 months.

Data from the NCR was used to determine the probability for breast cancer DM by T-stage and the time between surgical intervention and DM. To obtain a patient group with minimal risk of DM, we included only patients with complete removal of the tumor after surgical resection, relatively small tumors (T_1,2_) and no detectable metastasis (N_X_M_0_). The NCR recorded data for DM five years after surgical intervention. From the time to DM of 32 ± 18 months, we determined a *DT* of 1.7 ± 0.9 months for DM; threefold faster than the *DT* of 5.7 months (range 2.0-11.2) determined from primary tumor imaging data. A DM with a *DT* of 5.7 months would lead to discovery of a DM 9.5 years after initiation of the DM. Our 5-year (60 month) observation window is too short to observe tumors with a *DT* of 5.7 months. It is likely that our estimate of 1.7 months represents tumors with aggressive growth rates. Concurrent, the 5-year observation window may select for specific organs, because aggressive growth rates are more likely in organs that provide high levels of nutrients and tumor specific growth factors. Approximately three quarters of recurrences take place in the first five years [59]. With a 15-year observation window we expect to find a doubling time of 2.7 months. In addition, the literature value for *DT* of 5.7 months is determined on primary tumors, while the model fit *DT* of 1.7 months is determined on the DM. The DM may have a different *DT* than the primary lesion in the same patient due to natural selection in the metastatic cascade, differences in the tumor microenvironment or accumulation of growth enhancing mutations.

From murine studies, we conclude that dissemination rate is linearly dependent on the diameter of a lesion. For a diameter of 8 mm (typical T_1B_) we find a dissemination rate of 280 CTC/h · g tumor (range 90–470) when we fit the clinical data to our model. This is on the low end of the range of dissemination rates determined from the tumor efferent vein in human studies of 90–78,000 CTC/h · g tumor (Additional file 1: Table S3). Dissemination rates determined in murine models span a very wide range of 7 orders of magnitude (0.15-8,700,000 CTC/h · g tumor, Additional file 1: Table S2). While this variation may be caused by differences in the detection methods used or differences between cell lines, the variation between murine estimates makes comparison with our model futile.

Metastatic efficiency in our model is estimated at 1 metastasis per 60 million disseminated tumor cells. This is substantially less efficient than the murine model median estimate of 1 metastasis in 14,000 disseminated cells (range 1 in 170 to 1 in 1 million). The large difference of metastatic efficiency between murine model and human model may be attributed to many factors, including use of cell lines with high metastatic efficiency, the 2,000-fold difference in size between human and mouse and the immunodeficiency of most mouse models. A host specific (immune) response to tumor cells most likely reduces metastatic efficiency, and may reduce tumor growth of small lesions. Studies quantifying the impact of the host response on tumor growth are needed before inclusion in any model. Murine models suggest that disseminated cells have high survival in circulation and are efficient at extravasation, Additional file 1: Table S4. Survival of extravasated cells beyond 2 weeks is estimated between 4% and 50%, if these tumor cells continue to survive this would leave a substantial number of dormant cells scattered throughout the body, up to a million cells in our model, Additional file 1: Supplemental S4. These cells may constitute a malignant time-bomb, since dormant cells may be reactivated at a later time [60]. In the shorter term, metastatic efficiency is limited primarily by the ability of a disseminated cell to grow in a new site Table 4.

**Table 4:** **Blood flow to different organ systems**[61]**and % of breast cancer patients (N = 432) with distant metastases in these organs at time of death**[62]

**Organ**	**Cardiac output (%)**	**Px w/mets (%)**	**% Px w mets/% cardiac output**
Adrenal gl.	< 1	9	>9
Pleura	< 1	9	>9
Bone	5	12	2.40
Skin	9	7	0.78
Liver	27 ^a^	13	0.48
Brain	13	2	0.15
Lung	100	13	0.13
Kidney	20	2	0.10
Muscle	15	0	0
Other	10	< 3	< 0.3

Included in the table are all organs which receive at least 5% of cardiac output, or in which metastases were found at autopsy in at least 4% of patients. Px w mets = patients with metastasis.

^a^ 21% via portal vein, 6% direct.

Based on murine studies in different organs (see Additional file 1) we expect the model to be applicable to other cancers. It should be noted that tumors with high metastatic efficiency, such as melanoma [63] or non-small cell lung cancer [64] will have substantially lower numbers of CTC. Similarly, colorectal CTC are captured in the hepatic microcirculation and are lower when detected in the peripheral circulation [9,45]. Determination of tumor size is more difficult for some tumor types such as prostate cancer, which will result in higher error margins in the model parameters.

To determine the probability of metastases in a patient, three parameters are relevant, the dissemination rate, the growth rate and the metastatic efficiency. The dissemination rate can be determined from the CTC concentration, the growth rate and metastatic efficiency can be estimated from the primary tumor or, alternatively, by genotyping captured CTC. This is supported by the observation that both CTC concentration and hormone receptor status from primary tissue information are independent prognostic data in multivariate analyses [65,66].

The model can be applied to estimate the probability of metastases as a function of primary tumor size. Figure 3 illustrates that the model reasonably predicts the probability of DM for stages T_1B_ to T_2_. The probability of DM grows slightly faster in the data than in the model, which may be caused by a slow increase in dissemination rate or metastatic efficiency over time. With current imaging technology, 94% of detected lesions have a size of 6 mm or more, with a specificity of 40% [67]. From the data of the NCR, we conclude that current clinical practice in the Netherlands has similar detection characteristics, with 95% of the tumors detected when the tumor is 5 mm or larger, with a median size of 17 mm. The larger probability of DM for T_1A_ than T_1B_ in the NCR data is unexpected and raises the question whether these small tumors are truly more aggressive, or whether the difficulty to detect tumors smaller than 5 mm has caused a sampling bias in the T_1A_ sample.

To implement CTC as a screening tool, the improved CTC detection will need to have a minimal impact on the screened patient and to have similar specificity to radiological imaging. We note that by definition, CTC enumeration will not detect benign lesions. On the other hand, CTC detection could have excellent sensitivity and specificity for malignant lesions if the malignancy of detected CTC is confirmed with for example whole genome comparative genome hybridization [68,69].

## Conclusions

A model was developed to estimate tumor size and CTC concentration before distant metastasis occur. To reduce the overall probability of DM from 9.2% to 1% the tumor needs to be detected by the time it reaches 2.7 ± 1.6 mm. Clinical proof of this estimate requires an improvement in imaging technology that allows routine diagnosis of tumors smaller than 2.7 mm without a decrease in specificity of tumor detection. Alternatively, to achieve probability of DM of 1%, a tumor would need to be detected when the CTC concentration is 9 ± 6 CTC/L of whole blood. This requires at least a 15-fold improvement in the CTC detection limit. Subtypes of breast cancer with higher growth rate, higher metastatic efficiency, or higher dissemination rate would affect this estimate, requiring a smaller lesion, or a lower number of CTC, to be detected to achieve the overall probability of DM of 1%.

## Abbreviations

DM: Distant metastasis; CTC: Circulating tumor cells; DT: Doubling time of tumor; NCR: Netherlands cancer registry.

## Competing interests

This work was supported by Veridex LLC. Prof. Leon WMM Terstappen is an inventor of several patents related to the CTC technology that have been assigned to Veridex LLC, he is presently a consultant for Veridex and receives research funding from Veridex LLC. All remaining authors have declared no competing interest.

## Authors’ contributions

FC and LT designed the study and drafted the manuscript. FC and SB performed the statistical analysis. FC, SB and LT performed the data analysis and data interpretation. All authors read and approved the final manuscript.

## Pre-publication history

The pre-publication history for this paper can be accessed here:

http://www.biomedcentral.com/1471-2407/13/283/prepub

## Supplementary Material

Additional file 1 Detection of cancer before metastasis. Click here for file

## References

[B1] Minn AJ Massagué J Invasion and metastasis 2008 8 Philadelphia: Lippincott

[B2] Klein CA Cancer. The metastasis cascade Science 2008 321 5897 1785 1787 10.1126/science.1164853 18818347

[B3] Pantel K Brakenhoff RH Dissecting the metastatic cascade Nat Rev Cancer 2004 4 6 448 456 10.1038/nrc1370 15170447

[B4] Woodhouse EC Chuaqui RF Liotta LA General mechanisms of metastasis Ann Ny Acad Sci 1997 80 8 Suppl 1529 1537 10.1002/(sici)1097-0142(19971015)80:8+<1529::aid-cncr2>3.3.co;2-# 9362419

[B5] Fidler IJ Critical determinants of cancer metastasis: rationale for therapy Cancer Chemother Pharmacol 1999 43 Suppl S3 S10 10357552 10.1007/s002800051091

[B6] Weiss L Random and nonrandom processes in metastasis, and metastatic inefficiency Invasion Metastasis 1983 3 4 193 207 6588046

[B7] Chambers AF Groom AC MacDonald IC Dissemination and growth of cancer cells in metastatic sites Nat Rev Cancer 2002 2 8 563 572 10.1038/nrc865 12154349

[B8] de Bono JS Scher HI Montgomery RB Parker C Miller MC Tissing H Doyle GV Terstappen LWWM Pienta KJ Raghavan D Circulating tumor cells predict survival benefit from treatment in metastatic castration-resistant prostate cancer Clin Cancer Res 2008 14 19 6302 6309 10.1158/1078-0432.CCR-08-0872 18829513

[B9] Cohen SJ Punt CJA Iannotti N Saidman BH Sabbath KD Gabrail NY Picus J Morse M Mitchell E Miller MC Relationship of circulating tumor cells to tumor response, progression-free survival, and overall survival in patients with metastatic colorectal cancer J Clin Oncol 2008 26 19 3213 3221 10.1200/JCO.2007.15.8923 18591556

[B10] Cristofanilli M Budd GT Ellis MJ Stopeck A Matera J Miller MC Reuben JM Doyle GV Allard WJ Terstappen LW Circulating tumor cells, disease progression, and survival in metastatic breast cancer N Engl J Med 2004 351 8 781 791 10.1056/NEJMoa040766 15317891

[B11] Coumans FA Ligthart ST Uhr JW Terstappen LW Challenges in the enumeration and phenotyping of CTC Clin Cancer Res 2012 18 20 5711 5718 10.1158/1078-0432.CCR-12-1585 23014524

[B12] Rack BSC Andergassen U Lorenz R Zwingers T Schneeweiss A Lichtenegger W Beckmann MW Sommer H Pantel K Friese K Janni W Prognostic relevance of circulating tumor cells in the peripheral blood of primary breast cancer patients SABC 2010 S5 S6

[B13] Franken B de Groot MR Mastboom WJ Vermes I van der Palen J Tibbe AG Terstappen LW Circulating tumor cells, disease recurrence and survival in newly diagnosed breast cancer Breast Cancer Res 2012 14 5 R133 10.1186/bcr3333 23088337 PMC4053111

[B14] Lucci A Hall CS Lodhi AK Bhattacharyya A Anderson AE Xiao L Bedrosian I Kuerer HM Krishnamurthy S Circulating tumour cells in non-metastatic breast cancer: a prospective study Lancet Oncol 2012 13 7 688 695 10.1016/S1470-2045(12)70209-7 22677156

[B15] Pierga JY Hajage D Bachelot T Delaloge S Brain E Campone M Dieras V Rolland E Mignot L Mathiot C High independent prognostic and predictive value of circulating tumor cells compared with serum tumor markers in a large prospective trial in first-line chemotherapy for metastatic breast cancer patients Ann Oncol 2012 23 3 618 624 10.1093/annonc/mdr263 21642515

[B16] Gray JW Evidence emerges for early metastasis and parallel evolution of primary and metastatic tumors Cancer Cell 2003 4 1 4 6 10.1016/S1535-6108(03)00167-3 12892707

[B17] Klein CA The systemic progression of human cancer: a focus on the individual disseminated cancer cell–the unit of selection Adv Cancer Res 2003 89 35 67 14587870 10.1016/s0065-230x(03)01002-9

[B18] Schmidt-Kittler O Ragg T Daskalakis A Granzow M Ahr A Blankenstein TJF Kaufmann M Diebold J Arnholdt H Müller P From latent disseminated cells to overt metastasis: genetic analysis of systemic breast cancer progression Proc Natl Acad Sci 2003 100 13 7737 7742 10.1073/pnas.1331931100 12808139 PMC164657

[B19] Spratt JA von Fournier D Spratt JS Weber EE Decelerating growth and human breast cancer Ann Ny Acad Sci 1993 71 6 2013 2019 10.1002/1097-0142(19930315)71:6<2013::aid-cncr2820710615>3.0.co;2-v 8443753

[B20] Schmidt CM Settle SL Keene JL Westlin WF Nickols GA Griggs DW Characterization of spontaneous metastasis in an aggressive breast carcinoma model using flow cytometry Clin Exper Meta 1999 17 6 537 544 10.1023/A:1006719800907 10763921

[B21] Galanzha EI Shashkov EV Spring PM Suen JY Zharov VP *In vivo*, noninvasive, label-free detection and eradication of circulating metastatic melanoma cells using two-color photoacoustic flow cytometry with a diode laser Cancer Res 2009 69 20 7926 10.1158/0008-5472.CAN-08-4900 19826056 PMC2828368

[B22] Glaves D Correlation between circulating cancer cells and incidence of metastases Br J Cancer 1983 48 5 665 10.1038/bjc.1983.248 6639858 PMC2011528

[B23] Liotta LA Kleinerman J Saidel GM Quantitative relationships of intravascular tumor cells, tumor vessels, and pulmonary metastases following tumor implantation Cancer Res 1974 34 5 997 1004 4841969

[B24] Shaeffer J El-Mahdi AM Constable WC Radiation control of microscopic pulmonary metastases in C3H mice Ann Ny Acad Sci 1973 32 2 346 351 10.1002/1097-0142(197308)32:2<346::aid-cncr2820320210>3.0.co;2-0 4722916

[B25] Milas L Hunter N Withers HR Corynebacterium granulosum-induced protection against artificial pulmonary metastases of a syngeneic fibrosarcoma in mice Cancer Res 1974 34 3 613 620 4590922

[B26] Fidler IJ The relationship of embolic homogeneity, number, size and viability to the incidence of experimental metastasis Eur J Cancer 1973 9 3 223 227 4787857 10.1016/s0014-2964(73)80022-2

[B27] Lundgren B Observations on growth rate of breast carcinomas and its possible implications for lead time Ann Ny Acad Sci 1977 40 4 1722 1725 10.1002/1097-0142(197710)40:4<1722::aid-cncr2820400448>3.0.co;2-2 907980

[B28] Heuser L Spratt JS Polk HC Jr Growth rates of primary breast cancers Ann Ny Acad Sci 1979 43 5 1888 1894 10.1002/1097-0142(197905)43:5<1888::aid-cncr2820430545>3.0.co;2-m 445375

[B29] von Fournier D Weber E Hoeffken W Bauer M Kubli F Barth V Growth rate of 147 mammary carcinomas Ann Ny Acad Sci 1980 45 8 2198 2207 10.1002/1097-0142(19800415)45:8<2198::aid-cncr2820450832>3.0.co;2-7 7370960

[B30] Galante E Guzzon A Gallus G Mauri M Bono A De Carli A Merson M Di Pietro S Prognostic significance of the growth rate of breast cancer: preliminary evaluation on the follow-up of 196 breast cancers Tumori 1981 67 4 333 340 7314261 10.1177/030089168106700410

[B31] Tabbane F Bahi J Rahal K el May A Riahi M Cammoun M Hechiche M Jaziri M Mourali N Inflammatory symptoms in breast cancer. Correlations with growth rate, clinicopathologic variables, and evolution Ann Ny Acad Sci 1989 64 10 2081 2089 10.1002/1097-0142(19891115)64:10<2081::aid-cncr2820641019>3.0.co;2-7 2804897

[B32] Kuroishi T Tominaga S Morimoto T Tashiro H Itoh S Watanabe H Fukuda M Ota J Horino T Ishida T Tumor growth rate and prognosis of breast cancer mainly detected by mass screening Jpn J Cancer Res 1990 81 5 454 462 10.1111/j.1349-7006.1990.tb02591.x 2116393 PMC5918060

[B33] Peer PG van Dijck JA Hendriks JH Holland R Verbeek AL Age-dependent growth rate of primary breast cancer Ann Ny Acad Sci 1993 71 11 3547 3551 10.1002/1097-0142(19930601)71:11<3547::aid-cncr2820711114>3.0.co;2-c 8490903

[B34] Tilanus-Linthorst MM Kriege M Boetes C Hop WC Obdeijn IM Oosterwijk JC Peterse HL Zonderland HM Meijer S Eggermont AM Hereditary breast cancer growth rates and its impact on screening policy Eur J Cancer 2005 41 11 1610 1617 10.1016/j.ejca.2005.02.034 15978801

[B35] Weedon-Fekjaer H Lindqvist BH Vatten LJ Aalen OO Tretli S Breast cancer tumor growth estimated through mammography screening data Breast Cancer Res 2008 10 3 R41 10.1186/bcr2092 18466608 PMC2481488

[B36] Millet I Bouic-Pages E Hoa D Azria D Taourel P Growth of breast cancer recurrences assessed by consecutive MRI BMC Cancer 2011 11 155 10.1186/1471-2407-11-155 21527002 PMC3114791

[B37] Dent R Trudeau M Pritchard KI Hanna WM Kahn HK Sawka CA Lickley LA Rawlinson E Sun P Narod SA Triple-negative breast cancer: clinical features and patterns of recurrence Clin Cancer Res 2007 13 15 4429 4434 10.1158/1078-0432.CCR-06-3045 17671126

[B38] Butler TP Gullino PM Quantitation of cell shedding into efferent blood of mammary adenocarcinoma Cancer Res 1975 35 3 512 1090362

[B39] Swartz MA Kristensen CA Melder RJ Roberge S Calautti E Fukumura D Jain RK Cells shed from tumours show reduced clonogenicity, resistance to apoptosis, and *in vivo* tumorigenicity Br J Cancer 1999 81 5 756 759 10.1038/sj.bjc.6690760 10555742 PMC2374305

[B40] Wyckoff JB Jones JG Condeelis JS Segall JE A critical step in metastasis: *in vivo* analysis of intravasation at the primary tumor Cancer Res 2000 60 9 2504 10811132

[B41] Eliane JP Repollet M Luker KE Brown M Rae JM Dontu G Schott AF Wicha M Doyle GV Hayes DF Monitoring serial changes in circulating human breast cancer cells in murine xenograft models Cancer Res 2008 68 14 5529 10.1158/0008-5472.CAN-08-0630 18632603 PMC2789403

[B42] Goodale D Phay C Postenka CO Keeney M Allan AL Characterization of tumor cell dissemination patterns in preclinical models of cancer metastasis using flow cytometry and laser scanning cytometry Cytometry Part A 2009 75 4 344 355 10.1002/cyto.a.20657 18855920

[B43] Chambers AF MacDonald IC Schmidt EE Koop S Morris VL Khokha R Groom AC Steps in tumor metastasis: new concepts from intravital videomicroscopy Cancer Metastasis Rev 1995 14 4 279 301 10.1007/BF00690599 8821091

[B44] Glaves D Huben RP Weiss L Haematogenous dissemination of cells from human renal adenocarcinomas Br J Cancer 1988 57 1 32 35 10.1038/bjc.1988.4 3279993 PMC2246676

[B45] Wind J Tuynman JB Tibbe AGJ Swennenhuis JF Richel DJ van Berge Henegouwen MI Bemelman WA Circulating tumour cells during laparoscopic and open surgery for primary colonic cancer in portal and peripheral blood European J Surg Oncol (EJSO) 2009 35 9 942 950 10.1016/j.ejso.2008.12.003 19153024

[B46] Gruvberger S Ringnér M Chen Y Panavally S Saal LH Borg Å Fernö M Peterson C Meltzer PS Estrogen receptor status in breast cancer is associated with remarkably distinct gene expression patterns Cancer Res 2001 61 16 5979 5984 11507038

[B47] Cronin M Pho M Dutta D Stephans JC Shak S Kiefer MC Esteban JM Baker JB Measurement of gene expression in archival paraffin-embedded tissues: development and performance of a 92-gene reverse transcriptase-polymerase chain reaction assay Am J Pathol 2004 164 1 35 42 10.1016/S0002-9440(10)63093-3 14695316 PMC1602211

[B48] van't Veer LJ Dai H Van De Vijver MJ He YD Hart AA Mao M Peterse HL van der Kooy K Marton MJ Witteveen AT Gene expression profiling predicts clinical outcome of breast cancer Nature 2002 415 6871 530 536 10.1038/415530a 11823860

[B49] Perou CM Sørlie T Eisen MB van de Rijn M Jeffrey SS Rees CA Pollack JR Ross DT Johnsen H Akslen LA Molecular portraits of human breast tumours Nature 2000 406 6797 747 752 10.1038/35021093 10963602

[B50] Foekens JA Atkins D Zhang Y Sweep FC Harbeck N Paradiso A Cufer T Sieuwerts AM Talantov D Span PN Multicenter validation of a gene expression–based prognostic signature in lymph node–negative primary breast cancer J Clin Oncol 2006 24 11 1665 1671 10.1200/JCO.2005.03.9115 16505412

[B51] West M Blanchette C Dressman H Huang E Ishida S Spang R Zuzan H Olson JA Marks JR Nevins JR Predicting the clinical status of human breast cancer by using gene expression profiles Proc Natl Acad Sci 2001 98 20 11462 11467 10.1073/pnas.201162998 11562467 PMC58752

[B52] Braun S Pantel K Muller P Janni W Hepp F Kentenich CRM Gastroph S Wischnik A Dimpfl T Kindermann G Cytokeratin-positive cells in the bone marrow and survival of patients with stage I, II, or III breast cancer N Engl J Med 2000 342 8 525 533 10.1056/NEJM200002243420801 10684910

[B53] Braun S Vogl FD Naume B Janni W Osborne MP Coombes RC Schlimok G Diel IJ Gerber B Gebauer G A pooled analysis of bone marrow micrometastasis in breast cancer N Engl J Med 2005 353 8 793 802 10.1056/NEJMoa050434 16120859

[B54] Pierga JY Bidard FC Mathiot C Brain E Delaloge S Giachetti S de Cremoux P Salmon R Vincent-Salomon A Marty M Circulating tumor cell detection predicts early metastatic relapse after neoadjuvant chemotherapy in large operable and locally advanced breast cancer in a phase II randomized trial Clin Cancer Res 2008 14 21 7004 7010 10.1158/1078-0432.CCR-08-0030 18980996

[B55] Meng SD Tripathy D Frenkel EP Shete S Naftalis EZ Huth JF Beitsch PD Leitch M Hoover S Euhus D Circulating tumor cells in patients with breast cancer dormancy Clin Cancer Res 2004 10 24 8152 8162 10.1158/1078-0432.CCR-04-1110 15623589

[B56] Terstappen L Rao C Gross S Weiss AJ Peripheral blood tumor cell load reflects the clinical activity of the disease in patients with carcinoma of the breast Int J Oncol 2000 17 3 573 578 10938400 10.3892/ijo.17.3.573

[B57] Curtis C Shah SP Chin SF Turashvili G Rueda OM Dunning MJ Speed D Lynch AG Samarajiwa S Yuan Y The genomic and transcriptomic architecture of 2,000 breast tumours reveals novel subgroups Nature 2012 486 7403 346 352 22522925 10.1038/nature10983 PMC3440846

[B58] Wang Y Klijn JGM Zhang Y Sieuwerts AM Look MP Yang F Talantov D Timmermans M Meijer-van Gelder ME Yu J Gene-expression profiles to predict distant metastasis of lymph-node-negative primary breast cancer Lancet 2005 365 9460 671 679 15721472 10.1016/S0140-6736(05)17947-1

[B59] Clarke M Collins R Darby S Davies C Elphinstone P Evans E Godwin J Gray R Hicks C James S Effects of radiotherapy and of differences in the extent of surgery for early breast cancer on local recurrence and 15-year survival: an overview of the randomised trials Lancet 2005 366 9503 2087 16360786 10.1016/S0140-6736(05)67887-7

[B60] Uhr JW Pantel K Controversies in clinical cancer dormancy Proc Natl Acad Sci 2011 108 30 12396 10.1073/pnas.1106613108 21746894 PMC3145712

[B61] Sherwood L Human physiology: from cells to systems 1997 3 Belmont, CA: Wadsworth

[B62] Disibio G French SW Metastatic patterns of cancers - results from a large autopsy study Arch Pathol Lab Med 2008 132 6 931 939 18517275 10.5858/2008-132-931-MPOCRF

[B63] Rao C Bui T Connelly M Doyle G Karydis I Middleton MR Clack G Malone M Coumans FA Terstappen LW Circulating melanoma cells and survival in metastatic melanoma Int J Oncol 2011 38 3 755 760 21206975 10.3892/ijo.2011.896

[B64] Krebs MG Sloane R Priest L Lancashire L Hou JM Greystoke A Ward TH Ferraldeschi R Hughes A Clack G Evaluation and prognostic significance of circulating tumor cells in patients with non-small-cell lung cancer J Clin Oncol 2011 29 12 1556 1563 10.1200/JCO.2010.28.7045 21422424

[B65] Molloy TJ Bosma AJ Baumbusch LO Synnestvedt M Borgen E Russnes HG Schlichting E Van’t Veer LJ Naume B The prognostic significance of tumor cell detection in the peripheral blood versus the bone marrow in 733 early-stage breast cancer patients Breast Cancer Res 2011 13 R61 10.1186/bcr2898 21672237 PMC3218950

[B66] Saloustros E Perraki M Apostolaki S Kallergi G Xyrafas A Kalbakis K Agelaki S Kalykaki A Georgoulias V Mavroudis D Cytokeratin-19 mRNA-positive circulating tumor cells during follow-up of patients with operable breast cancer: prognostic relevance for late relapse Breast Cancer Res 2011 13 3 R60 10.1186/bcr2897 21663668 PMC3218949

[B67] Kelly KM Dean J Comulada WS Lee SJ Breast cancer detection using automated whole breast ultrasound and mammography in radiographically dense breasts Eur Radiol 2010 20 3 734 742 10.1007/s00330-009-1588-y 19727744 PMC2822222

[B68] Klein CA Seidl S Petat-Dutter K Offner S Geigl JB Schmidt-Kittler O Wendler N Passlick B Huber RM Schlimok G Combined transcriptome and genome analysis of single micrometastatic cells Nat Biotechnol 2002 20 4 387 392 10.1038/nbt0402-387 11923846

[B69] Stoecklein NH Hosch SB Bezler M Stern F Hartmann CH Vay C Siegmund A Scheunemann P Schurr P Knoefel WT Direct genetic analysis of single disseminated cancer cells for prediction of outcome and therapy selection in esophageal cancer Cancer Cell 2008 13 5 441 453 10.1016/j.ccr.2008.04.005 18455127

