# Supplementary material for: Detection of cancer before distant metastasis
Source: BMC Cancer. 2013 Jun 13;13:283. doi: 10.1186/1471-2407-13-283 (PMC3684526; doi:10.1186/1471-2407-13-283)
Supplement: Additional file 1 — Detection of cancer before metastasis. [file 1471-2407-13-283-S1.docx]

Detection of cancer before metastasis

**Additional file 1: Supplemental S1. Metastatic cascade model derivation**

The steps in the metastatic cascade are summarized in figure 1. A tumor grows locally (1). Cells disseminate from the primary tumor (2). The tumor cells that survive in the circulation (3) ultimately arrest in the microcirculation of an organ and may extravasate into the surrounding tissue (4). Extravasated cells can either; survive as a singular dormant cell (5A), form a micro-metastasis (5B), or grow into a macro-metastasis (5C).

*1: Local growth:* Functions described for tumor growth are exponential [[1-8](#_ENREF_1)], Gompertz [[8](#_ENREF_8), [9](#_ENREF_9)] or logistic function [[8](#_ENREF_8), [10](#_ENREF_10), [11](#_ENREF_11)]. Gompertz and logistic functions have a slowing growth rate as the tumor reaches a maximum size *N_max_* (N = number of tumor cells) at a certain time (*t*). *N_max_* is typically chosen at 10^12^ cells / 1 kilogram. Based on a comparison of possible functions across a wide range of breast tumor sizes [[8](#_ENREF_8)] we use the logistic equation:

$N_{mass}\left( t \right)=\frac{N_{max}}{\left[ 1+\left( N_{max}^{1/4}-1 \right)e^{{-ln\left( 2 \right) t}/{4 DT}} \right]^{4}}\approx\frac{N_{max}}{\left[ 1+\left( {N_{max}e}^{{-ln\left( 2 \right) t}/{DT}} \right)^{1/4} \right]^{4}}$ (S1)

The time needed for a tumor to double in size, the doubling time (*DT*), changes as the tumor grows. In equation S1 DT at time t (DT_t_) is related to DT at time 0 [[11](#_ENREF_11)]:

${DT}_{t}=-\frac{4 DT}{ln\left( 2 \right)}ln\left( \frac{\sqrt[4]{\left( {N_{max}}/{2N_{mass}(t)} \right)} -1}{\sqrt[4]{\left( {N_{max}}/{N_{mass}(t)} \right)}-1} \right)$ (S2)

In a model with growth slowing over time DT is typically determined for a tumor size of 12 mm (table 2), at this size DT is 23% longer compared to the DT at the start of growth, and at a size of 8 mm DT is 16% longer. We assume macro-metastases grow according to equation 1 and have the same doubling time as the primary. Changes in growth rate as a function of supply of nutrition, due to occurrence of growth enhancing mutations or due to chemo or hormonal therapy are not considered in any of the growth models.

*2. Dissemination to circulation:* The relationship between tumor diameter (*D_mass_*) and the number of disseminated cells (*N_diss_*) is assumed linear with coefficient C_diss_ and is derived from murine data comparing CTC counts to the diameter of the primary tumor [[12-15](#_ENREF_12)]. To derive the diameter of the lesion from the number of cells (*N_mass_*), we assumed a spherical lesion. The primary tumor disseminates cells into the bloodstream at a certain rate (*R_diss_*,) as described in equation S3:

$R_{diss}= C_{diss}\cdot D_{mass}$ (S3)

*3. Survival in circulation:* Disseminated cells $(N_{diss})$ will have a probability that they can survive in circulation ${(P}_{surv circ})$ a precondition to form distant metastasis. The number of surviving cells ${(N}_{surv circ} )$is defined as :

$N_{surv circ} =N_{diss}\cdot P_{surv circ}$ (S4)

*4. Extravasation:* The ability to extravasate is also a condition for formation of a distant metastasis. The number of tumor cells that extravasate into the tissue ($N_{ex vas}$) can be defined as:

$N_{ex vas} =N_{surv circ}\cdot P_{ex vas}$ (S5)

$P_{ex vas}$ in this equation is the probability that a tumor cell extravasates. The fate of a tumor cell once it has extravasated is death, dormancy, or growth into a micro- or macro-metastatic site depicted as step 5 in figure 1.

*5A. Dormancy:* An extravasated cell may survive in the new micro environment, but cease to grow; the number of dormant cells ${(N}_{dorm}) are defined as$:

$N_{dorm}=N_{ex vas}\cdot P_{surv dorm}$ (S6)

$P_{surv dorm}$ in this equation is the probability that a tumor cell remains dormant

*5B. Micro-metastasis:* An extravasated cell may replicate briefly or very slowly to form a micro-metastasis the number of tumor cells that form a micro-metastasis ${(N}_{micro met})$are defined as:

$N_{micro met}=N_{ex vas}\cdot P_{micro met}$ (S7)

$P_{micro met}$ in this equation is the probability that a tumor cell forms a micro-metastasis

*5C. Macro-metastasis:* An extravasated cell may continue to replicate rapidly and form a macro-metastasis. The number of tumor cells that form a macro-metastasis ${(N}_{macro met})$are defined as:

$N_{macro met}=N_{ex vas}\cdot P_{macro met}$ (S8)

$P_{macro met}$is the probability that a tumor cell forms a macro-metastasis. The macro-metastases are the most unfavorable outcome, posing the most immediate threat to survival of the patient. The total number of macro-metastases is found by integrating over time:

$$N_{total macro met}=\int R_{diss}\cdot P_{surv circ}\cdot P_{ex vas}\cdot P_{macro met}dt$$

$$=\int{\gamma_{metastatic}\cdot R}_{diss}dt$$

$=\gamma_{metastatic}{\cdot C}_{diss}\int D_{mass}dt$ (S9)

Where the metastatic efficiency and the dissemination coefficient are taken out of the integral because they are assumed time independent. Metastatic efficiency is defined as:

$\gamma_{metastatic}=P_{surv circ}\cdot P_{ex vas}\cdot P_{macro met}$ (S10)

Equations S4-S6 and S8 provide a linear relationship between the number of cells injected into the circulation and the number of macro-metastases, as suggested in literature [[16-18](#_ENREF_16)]. The number of metastases formed, equation 3/S9, is equal to the total number of cells disseminated from the tumor, times the product of probabilities that this cell survives in the circulation, extravasates, progresses to a macro-metastasis. The metastatic efficiency of the tumor (𝛾_metastatic_) is the probability that a cell that entered the circulation forms a distant metastasis. The number of disseminated cells (*N_diss_*) is measurable by detecting the number of CTC, while the metastatic efficiency (𝛾_metastatic_) may be measurable either by genotyping these CTC or the primary tissue.

**Additional file 1: Supplemental S2. CTC concentration and capture of CTC by the microvasculature**

Cells that disseminate from the primary tumor are present in a high concentration in the efferent vein, and are ultimately diluted by the whole blood volume. First the concentration of CTC from the tumor efferent vein (*[CTC]_efferent_*) is given, with the number of CTC disseminated (*R_diss_*) independent of the local flow rate [[19](#_ENREF_19)]:

$\left[ CTC \right]_{efferent}=\frac{R_{diss}}{Q_{efferent}}$ (S11)

The flow from the efferent vein (*Q_efferent_*) is mixed with the whole blood volume at which time the concentration becomes:

$\left[ CTC \right]=\frac{R_{diss}}{Q_{efferent}}\cdot\frac{Q_{efferent}}{Q_{total}}+{[CTC]}_{residual}=\frac{R_{diss}}{Q_{total}}{+ [CTC]}_{residual}$ (S12)

With *[CTC]_residual_* the concentration of CTC that have made more than one passage through the circulation. We assume this *[CTC]_residual_* to be 0 because we lack the required information to make a better estimate.

**Additional file 1: Supplemental S3. Individual probabilities in the metastatic cascade**

The metastatic efficiency is defined as the product of the probability of survival in circulation, the probability of extravasation, and the probability of growth into a macro-metastasis. Human studies for probabilities in the metastatic cascade are quite limited; we only found values for survival in circulation from studies that determined that 23-80% of CTC had caspase cleaved cytokeratin (M30-positive) and were thus undergoing apoptosis [[20](#_ENREF_20), [21](#_ENREF_21)], therefore survival in circulation was less than 80%. Considering that cytokeratin cleavage occurs late in apoptosis and the % necrotic cells was not determined, true survival in circulation is most likely lower.

To dissect the individual probabilities in the metastatic efficiency, intra-vital video microscopy (IVM) [[22](#_ENREF_22), [23](#_ENREF_23)] has been used extensively. Using IVM, The median estimate for probability of extravasation is 65%, table 3. Due to limitations in the time that a single animal can be observed, most studies monitored a time window of 24 hours. A single study [[24](#_ENREF_24)] monitored the process up to 72 hours, which found that while 55% of cells arrested in the microvasculature had extravasated by 24 hours, 96% had extravasated by 72 hours. It is therefore likely that the estimate of 65% is too low because the process takes longer than the typical observation window of up to 24 hours. Probability of extravasation was determined in organs with high incidence of metastases for all studies and may be lower in other organs. The probability of surviving in the circulation was derived together with the probability of extravasation, table 3, precluding direct determination of the probability of survival. The product of probabilities found ranges from 43% to 89% with a median at 80%. Using the estimate of 65% for extravasation, the probability of surviving in circulation is at least 70%.

An extravasated cell can die, become dormant, form a micro-metastasis or continue to grow into a macro-metastasis. IVM studies that compared tumor cell distribution in an organ after injection and 2-3 weeks later found that the probability of surviving as a single cell (dormant) was 36% (range 4%-50%, supplemental table S2. If we assume that 36% of extravasated cells continue to survive over the years, approximately 1·10^9^ dormant malignant cells have scattered throughout the body by the time of surgery. The probability of forming micro-metastases is estimated at 6% (range 1%-80%), supplemental table S2. The probability that an extravasated cell forms a macro-metastasis was estimated by IVM at 0.025% (range 0.001-6%), supplemental table S2.

The metastatic efficiency is the probability that a disseminated cell grows in a new site. The product of the probability of survival in circulation, extravasation and growth to a macro-metastasis is the metastatic efficiency. When we combine the IVM estimates for each probability together in equation S10, we find 𝛾_metastatic_ of 0.011%. Other methods which determined the metastatic efficiency from the number of metastases formed after injection of a known number of cells estimated 𝛾_metastatic_ at a comparable value of 0.005% (range 0.0001-6%) [[16-18](#_ENREF_16), [25-28](#_ENREF_25)].

This metastatic efficiency is limited primarily by the ability of a disseminated cell to grow in a new site. The reasons for the limited ability to grow in a new organ is still under investigation, potential causes include genetic predisposition of the disseminated cell, proximity to other tumor cells, and local microenvironment (growth factors, nutrients, space). Proximity of other cells is suggested from two experiments; 1) when clumps of 4-7 cells are injected versus the same total number of individual cells from the same population, the probability of forming a macro-metastasis is increased 3-10 fold [[18](#_ENREF_18), [29](#_ENREF_29)] and 2) migration to preferred sites of growth is observed after extravasation [[22](#_ENREF_22), [24](#_ENREF_24)], bringing tumor cells closer together. Genetic predisposition is suggested by a relatively high metastatic efficiency of tumor cells harvested from metastases of other tumors [[17](#_ENREF_17), [26](#_ENREF_26)]. In one study, the metastases from MDA-435 cells were collected and seeded to new animals. These cells were more likely to metastasize to the same organ again, indicating that de genetic makeup of a cell is important in determining where metastases form [[28](#_ENREF_28)].

**Additional file 1:Table S1: Metastatic cascade parameter estimates from our model, human and murine studies.** Literature values are the median of all estimates with the range of estimates in parenthesis. Detailed data for each publication is given in supplementary tables indicated in the right hand column

| parameter | symbol^a^ | model | human | murine | table |
| --- | --- | --- | --- | --- | --- |
| doubling time (months) | DT | 1.7± 0.9 | 5.7  (2.0-11.2) |  | 2 |
| dissemination rate (CTC/h/g) | R_diss_ | 280  (90-470) | 3.1 · 10^3^  (90 - 78 · 10^3^) | 1.0·10^5^  (1.5·10^-1^-8.7·10^6^) | 3,S2 |
| critical Size (g) | N_crit_ | < 1 |  | 0.4  (0.2-0.8) | S2 |
| survival in circulation | P_surv circ_ |  | < 0.8 | 90%  (70-95%) | S4 |
| extravasation | P_exvas_ |  |  | 65%  (20-96%) | S4 |
| dormant survival | P_surv dorm_ |  |  | 36%  (35-50%) | S3 |
| formation of micro met | P_micro met_ |  |  | 6%  (1-80%) | S3 |
| formation of macro met | P_macro met_ |  |  | 0.01%  (1.6·10^-4^-0.06) | S3 |
| metastatic efficiency | 𝛾_metastatic_ | 1.7·10^-8^  (1.3·10^-8^-4.2·10^-8^) |  | 7·10^-5^  (1·10^-6^-6·10^-3^) | S3 |

^a^ See Results and Supplemental S1 for further descriptions

**Additional file 1: Table S2: Murine tumor size and CTC dissemination rate.**

| Publication | Method ^a^ | Cell line | Tumor size (g) | CTC (/mL blood) | CTC/h/g tumor | N_crit_(g) |
| --- | --- | --- | --- | --- | --- | --- |
| Liotta 1974 [[15](#_ENREF_15)] | M/Eff | T241 (fibrosarcoma) | 0.5-3.6 | 2-21 | 0.1·10^3^-1.7·10^3^ | 0.4 |
| Butler 1975 [[30](#_ENREF_30)] | FM/Eff | MTW9 (murine breast) | 2.6-3.7 | 17·10^3^-20·10^3^ | 1.3·10^5^-1.7·10^5^ | - |
| Swartz 1999 [[19](#_ENREF_19)] | FCM/Eff | LS174T (colon) | 0.5 |  | 2.6·10^5^ | - |
|  |  | LS LiM 6 (colon) | 0.5 |  | 1.0·10^5^ |  |
| Schmidt 1999 [[12](#_ENREF_12)] | FCM/Tot | MDA-435-HAL-GFP (breast) | 0.4-3.1 | 3250-7000 | 1.5·10^6^ ‑15.8·10^6^ | 0.4 |
| Wyckoff 2000 [[31](#_ENREF_31)] | Cul/Tot | MTLn3 – GFP  MTC – GFP (murine breast) | 31.0  44.5 | 5.7  0.06 | 1.2·10^3^  0.15 | - |
| Eliane 2008 [[32](#_ENREF_32)] | FM/Tot | MDA-231 (breast)  SUM-159 (breast)  SKBR-3 (breast) | 0.2-4.4  0.7-6.1  0.1-3.3 | 106-975  0-335  0-121 | 4.8·10^4^-1.7·10^6^  0-7.2·10^4^  0-1.5·10^5^ | 0.2  0.8  0.3 |
| Goodale 2009 [[33](#_ENREF_33)] | FCM/Tot | MDA-435-HAL | 1.73 | 7790 | 4.3·10^6^ | - |

^a^ FCM: flowcytometry, FM: fluorescence microscopy, M: microscopy, Cul: culture of blood cells, Eff: measurement performed by directly perfusing the tumor bearing organ and collecting venous output, Tot: measurement performed by detecting CTC concentration in right heart, converted to shed rate by multiplying with cardiac output of animal (mouse 16 mL/min [[34](#_ENREF_34)], rat 110 mL/min [[35](#_ENREF_35)]). Assumes negligible number of cells make second pass through circulation.

**Additional file 1: Table S3: Cell fate after extravasation.** All probabilities in %

| Publication | Method ^a^ | Cell line | P_dorm_ ^b^ | P_micro met_ | P_macro met_ |
| --- | --- | --- | --- | --- | --- |
| Schaeffer 1973 [[16](#_ENREF_16)] | histology | C3HBA (murine breast) |  |  | 0.005 ^c^ |
| Fidler 1973 [[18](#_ENREF_18)] | histology | B16 (melanoma) |  |  | 0.6 ^c^ |
| Milas 1974 [[17](#_ENREF_17)] | autopsy | mammary carcinoma ^d^ |  |  | 0.03 ^c^ |
| Mayhew 1984 [[25](#_ENREF_25)] | histology | B16F10 (melanoma)  Lewis (lung) |  |  | 0.6  0.002 ^c^ |
| Price 1989 [[27](#_ENREF_27)] | histology | HT-29 (colon) |  |  | >0.0001 ^c^ |
| Price 1990 [[28](#_ENREF_28)] | autopsy | MDA-MB-435 |  |  | 0.007 ^c^ |
| Morris 1994 [[24](#_ENREF_24)] | IVM | D2A1  D2.OR (murine breast) |  | < 1 |  |
| Chambers 1995 [[22](#_ENREF_22)] | IVM | various | 50 |  |  |
| Luzzi 1998 [[36](#_ENREF_36)] | IVM | B16F1 (melanoma) | 36 | 2 | 0.025 |
| Naumov 1999 [[37](#_ENREF_37)] | IVM | CHO-K1-GFP (hamster ovary) |  | 80 |  |
| Cameron 2000 [[38](#_ENREF_38)] | IVM | B16F10 | 3.5 |  | 6 |
| Steinbauer 2003 [[39](#_ENREF_39)] | IVM | CT-26 (colon) |  | 6 |  |
| Mook 2003 [[40](#_ENREF_40)] | IVM | CC531s (murine colon) |  |  | 0.001 |
| Podsypanina 2008 [[26](#_ENREF_26)] | BL | spontaneous carcinoma ^d^ |  |  | 0.014 ^c^ |

^a^ IVM: intra vital fluorescence microscopy, BL: Bioluminescence, ^b^ survival as single cell > 2 weeks, ^c^ probability to form macro-metastasis from number of injected cells, ^d^ induced in one animal by feeding of a carcinogen, and then minced to inoculate another animal.

**Additional file 1: Table S4: Survival in circulation and extravasation probability as observed with intra-vital video microscopy**

| Publication | Cell line | Injection site | Observation organ | Time (h)^a^ | P_ex vas_  % | P_surv_^·^ P_ex vas_  % |
| --- | --- | --- | --- | --- | --- | --- |
| Morris 1994 [[24](#_ENREF_24)] | D2A1/D2.OR (murine breast) | mesentery | liver | 24  72 | 55  96 |  |
| Koop 1995 [[41](#_ENREF_41)] | B16F10 (melanoma) | chick embryo |  | 24 |  | >80 |
| Chambers 1995 [[22](#_ENREF_22)] | various ^b^ | various^b^ | various^b^ |  |  | 89 |
| Steinbauer 2003 [[39](#_ENREF_39)] | CT-26 (colon) | mesentery | liver | 48 |  | 43 |
| Schlutter 2006 [[42](#_ENREF_42)] | HT-29LMM (colon) | left heart | liver | 0.5 | 29 |  |
| Martin 2010 [[43](#_ENREF_43)] | R221A-GFP (breast) | spleen  tail | liver  lung | 24  24 | 50  20 |  |

^a^ observation time, ^b^ Review of other publications.

**Additional file 1**

1. Kuroishi T, Tominaga S, Morimoto T, Tashiro H, Itoh S, Watanabe H, Fukuda M, Ota J, Horino T, Ishida T *et al*: **Tumor growth rate and prognosis of breast cancer mainly detected by mass screening**. *Jpn J Cancer Res* 1990, **81**(5):454-462.

2. Tabbane F, Bahi J, Rahal K, el May A, Riahi M, Cammoun M, Hechiche M, Jaziri M, Mourali N: **Inflammatory symptoms in breast cancer. Correlations with growth rate, clinicopathologic variables, and evolution**. *Ann Ny Acad Sci* 1989, **64**(10):2081-2089.

3. Heuser L, Spratt JS, Polk HC, Jr.: **Growth rates of primary breast cancers**. *Ann Ny Acad Sci* 1979, **43**(5):1888-1894.

4. Galante E, Guzzon A, Gallus G, Mauri M, Bono A, De Carli A, Merson M, Di Pietro S: **Prognostic significance of the growth rate of breast cancer: preliminary evaluation on the follow-up of 196 breast cancers**. *Tumori* 1981, **67**(4):333-340.

5. Lundgren B: **Observations on growth rate of breast carcinomas and its possible implications for lead time**. *Ann Ny Acad Sci* 1977, **40**(4):1722-1725.

6. Peer PG, van Dijck JA, Hendriks JH, Holland R, Verbeek AL: **Age-dependent growth rate of primary breast cancer**. *Ann Ny Acad Sci* 1993, **71**(11):3547-3551.

7. Tilanus-Linthorst MM, Kriege M, Boetes C, Hop WC, Obdeijn IM, Oosterwijk JC, Peterse HL, Zonderland HM, Meijer S, Eggermont AM *et al*: **Hereditary breast cancer growth rates and its impact on screening policy**. *Eur J Cancer* 2005, **41**(11):1610-1617.

8. Spratt JA, von Fournier D, Spratt JS, Weber EE: **Decelerating growth and human breast cancer**. *Ann Ny Acad Sci* 1993, **71**(6):2013-2019.

9. von Fournier D, Weber E, Hoeffken W, Bauer M, Kubli F, Barth V: **Growth rate of 147 mammary carcinomas**. *Ann Ny Acad Sci* 1980, **45**(8):2198-2207.

10. Weedon-Fekjaer H, Lindqvist BH, Vatten LJ, Aalen OO, Tretli S: **Breast cancer tumor growth estimated through mammography screening data**. *Breast Cancer Res* 2008, **10**(3):R41.

11. Millet I, Bouic-Pages E, Hoa D, Azria D, Taourel P: **Growth of breast cancer recurrences assessed by consecutive MRI**. *BMC Cancer* 2011, **11**:155.

12. Schmidt CM, Settle SL, Keene JL, Westlin WF, Nickols GA, Griggs DW: **Characterization of spontaneous metastasis in an aggressive breast carcinoma model using flow cytometry**. *Clinical and Experimental Metastasis* 1999, **17**(6):537-544.

13. Galanzha EI, Shashkov EV, Spring PM, Suen JY, Zharov VP: **In vivo, noninvasive, label-free detection and eradication of circulating metastatic melanoma cells using two-color photoacoustic flow cytometry with a diode laser**. *Cancer Research* 2009, **69**(20):7926.

14. Glaves D: **Correlation between circulating cancer cells and incidence of metastases**. *British Journal of Cancer* 1983, **48**(5):665.

15. Liotta LA, Kleinerman J, Saidel GM: **Quantitative relationships of intravascular tumor cells, tumor vessels, and pulmonary metastases following tumor implantation**. *Cancer Research* 1974, **34**(5):997.

16. Shaeffer J, El‐Mahdi AM, Constable WC: **Radiation control of microscopic pulmonary metastases in C3H mice**. *Ann Ny Acad Sci* 1973, **32**(2):346-351.

17. Milas L, Hunter N, Withers HR: **Corynebacterium granulosum-induced protection against artificial pulmonary metastases of a syngeneic fibrosarcoma in mice**. *Cancer Research* 1974, **34**(3):613-620.

18. Fidler IJ: **The relationship of embolic homogeneity, number, size and viability to the incidence of experimental metastasis**. *Eur J Cancer* 1973, **9**(3):223-227.

19. Swartz MA, Kristensen CA, Melder RJ, Roberge S, Calautti E, Fukumura D, Jain RK: **Cells shed from tumours show reduced clonogenicity, resistance to apoptosis, and in vivo tumorigenicity**. *British Journal of Cancer* 1999, **81**(5):756-759.

20. Swennenhuis JF, Tibbe AGJ, Levink R, Sipkema RCJ, Terstappen LWMM: **Characterization of circulating tumor cells by fluorescence in situ hybridization**. *Cytometry Part A* 2009, **75A**(6):520-527.

21. Rossi E, Basso U, Celadin R, Zilio F, Pucciarelli S, Aieta M, Barile C, Sava T, Bonciarelli G, Tumolo S *et al*: **M30 Neoepitope Expression in Epithelial Cancer: Quantification of Apoptosis in Circulating Tumor Cells by CellSearch Analysis**. *Clinical Cancer Research* 2010, **16**(21):5233-5243.

22. Chambers AF, MacDonald IC, Schmidt EE, Koop S, Morris VL, Khokha R, Groom AC: **Steps in tumor metastasis: new concepts from intravital videomicroscopy**. *Cancer Metastasis Rev* 1995, **14**(4):279-301.

23. Beerling E, Ritsma L, Vrisekoop N, Derksen PWB, van Rheenen J: **Intravital microscopy: new insights into metastasis of tumors**. *J Cell Sci* 2011, **124**(3):299-310.

24. Morris VL, Koop S, MacDonald IC, Schmidt EE, Grattan M, Percy D, Chambers AF, Groom AC: **Mammary carcinoma cell lines of high and low metastatic potential differ not in extravasation but in subsequent migration and growth**. *Clinical & Experimental Metastasis* 1994, **12**(6):357-367.

25. Mayhew E, Glaves D: **Quantitation of tumorigenic disseminating and arrested cancer cells**. *British Journal of Cancer* 1984, **50**(2):159.

26. Podsypanina K, Du YCN, Jechlinger M, Beverly LJ, Hambardzumyan D, Varmus H: **Seeding and propagation of untransformed mouse mammary cells in the lung**. *Science* 2008, **321**(5897):1841.

27. Price JE, Daniels LM, Campbell DE, Giavazzi R: **Organ distribution of experimental metastases of a human colorectal carcinoma injected in nude mice**. *Clinical & Experimental Metastasis* 1989, **7**(1):55-68.

28. Price JE, Polyzos A, Zhang RD, Daniels LM: **Tumorigenicity and metastasis of human breast carcinoma cell lines in nude mice**. *Cancer Research* 1990, **50**(3):717-721.

29. Liotta LA, Saidel MG, Kleinerman J: **The significance of hematogenous tumor cell clumps in the metastatic process**. *Cancer Research* 1976, **36**(3):889-894.

30. Butler TP, Gullino PM: **Quantitation of cell shedding into efferent blood of mammary adenocarcinoma**. *Cancer Research* 1975, **35**(3):512.

31. Wyckoff JB, Jones JG, Condeelis JS, Segall JE: **A critical step in metastasis: in vivo analysis of intravasation at the primary tumor**. *Cancer Research* 2000, **60**(9):2504.

32. Eliane JP, Repollet M, Luker KE, Brown M, Rae JM, Dontu G, Schott AF, Wicha M, Doyle GV, Hayes DF: **Monitoring serial changes in circulating human breast cancer cells in murine xenograft models**. *Cancer Research* 2008, **68**(14):5529.

33. Goodale D, Phay C, Postenka CO, Keeney M, Allan AL: **Characterization of tumor cell dissemination patterns in preclinical models of cancer metastasis using flow cytometry and laser scanning cytometry**. *Cytometry Part A* 2009, **75**(4):344-355.

34. Janssen B, Debets J, Leenders P, Smits J: **Chronic measurement of cardiac output in conscious mice**. *American Journal of Physiology-Regulatory, Integrative and Comparative Physiology* 2002, **282**(3):R928-R935.

35. Delp MD, Evans MV, Duan C: **Effects of aging on cardiac output, regional blood flow, and body composition in Fischer-344 rats**. *J Appl Physiol* 1998, **85**(5):1813.

36. Luzzi KJ, MacDonald IC, Schmidt EE, Kerkvliet N, Morris VL, Chambers AF, Groom AC: **Multistep nature of metastatic inefficiency: dormancy of solitary cells after successful extravasation and limited survival of early micrometastases**. *The American Journal of Pathology* 1998, **153**(3):865.

37. Naumov GN, Wilson SM, MacDonald IC, Schmidt EE, Morris VL, Groom AC, Hoffman RM, Chambers AF: **Cellular expression of green fluorescent protein, coupled with high-resolution in vivo videomicroscopy, to monitor steps in tumor metastasis**. *J Cell Sci* 1999, **112**(12):1835.

38. Cameron MD, Schmidt EE, Kerkvliet N, Nadkarni KV, Morris VL, Groom AC, Chambers AF, MacDonald IC: **Temporal progression of metastasis in lung: cell survival, dormancy, and location dependence of metastatic inefficiency**. *Cancer Research* 2000, **60**(9):2541-2546.

39. Steinbauer M, Guba M, Cernaianu G, Kohl G, Cetto M, Kunz-Schughart LA, Geissler EK, Falk W, Jauch KW: **GFP-transfected tumor cells are useful in examining early metastasis in vivo, but immune reaction precludes long-term tumor development studies in immunocompetent mice**. *Clinical & Experimental Metastasis* 2003, **20**(2):135-141.

40. Mook OR, Van Marle J, Vreeling-Sindelarova H, Jonges R, Frederiks WM, Van Noorden CJ: **Visualization of early events in tumor formation of eGFP-transfected rat colon cancer cells in liver**. *Hepatology* 2003, **38**(2):295-304.

41. Koop S, MacDonald IC, Luzzi K, Schmidt EE, Morris VL, Grattan M, Khokha R, Chambers AF, Groom AC: **Fate of melanoma cells entering the microcirculation: over 80% survive and extravasate**. *Cancer Research* 1995, **55**(12):2520-2523.

42. Schluter K, Gassmann P, Enns A, Korb T, Hemping-Bovenkerk A, Holzen J, Haier J: **Organ-specific metastatic tumor cell adhesion and extravasation of colon carcinoma cells with different metastatic potential**. *The American Journal of Pathology* 2006, **169**(3):1064-1073.

43. Martin MD, Kremers GJ, Short KW, Rocheleau JV, Xu L, Piston DW, Matrisian LM, Gorden DL: **Rapid Extravasation and Establishment of Breast Cancer Micrometastases in the Liver Microenvironment**. *Molecular Cancer Research* 2010, **8**(10):1319-1327.
